# A detailed review of the spinal accessory nerve and its anatomical variations with cadaveric illustration

**DOI:** 10.1007/s12565-024-00770-w

**Published:** 2024-05-02

**Authors:** Siôn Owain Roberts, Arun Cardozo

**Affiliations:** 1 Department of Anatomy, Faculty of Medicine and Health, School of Medicine, University of Leeds, Leeds, Yorkshire LS2 9JT UK; 2 Department of Otorhinolaryngology, Lancashire Teaching Hospital NHS Trust, Sharoe Green Lane North, Fulwood, Preston, Lancashire PR2 9HT UK

**Keywords:** Accessory, Classification, Course, Nerve, Variation

## Abstract

The spinal accessory nerve, considered part of the eleventh cranial nerve, provides motor innervation to sternocleidomastoid and trapezius. A comprehensive literature review and two cadaveric dissections were undertaken. The spinal accessory nerve originates from the spinal accessory nucleus. Its rootlets unite and ascend between the denticulate ligament and dorsal spinal rootlets. Thereafter, it can anastomose with spinal roots, such as the McKenzie branch, and/or cranial roots. The spinal accessory nerve courses intracranially via foramen magnum and exits via jugular foramen, within which it usually lies anteriorly. Extracranially, it usually crosses anterior to the internal jugular vein and lies lateral to internal jugular vein deep to posterior belly of digastric. The spinal accessory nerve innervates sternocleidomastoid, receives numerous contributions in the posterior triangle and terminates within trapezius. Its posterior triangle course approximates a perpendicular bisection of the mastoid-mandibular angle line. The spinal accessory nerve contains sensory nociceptive fibres. Its cranial nerve classification is debated due to occasional non-fusion with the cranial root. Surgeons should familiarize themselves with the variable course of the spinal accessory nerve to minimize risk of injury. Patients with spinal accessory nerve injuries might require specialist pain management.

## Introduction

The accessory nerve is classified as the eleventh cranial nerve (Binder 2010; Netter 2006; Standring 2005). It was originally named because of its “accessory” cranial input (Hierons and Meyer 1962; Simon et al. 2011; Skinner 1961). It comprises the Spinal Accessory Nerve (SAN), providing motor innervation to sternocleidomastoid (SCM) and trapezius muscles, and a short cranial component, originating from the brainstem. This configuration has conventionally been taught to medical students for decades.

Its function is routinely tested in cranial nerve examinations by turning the head and shrugging the shoulders against resistance. Damage at any point along the nerve’s course results in functional deficit of SCM and trapezius. Pain and reduced range of shoulder motion post-injury can render routine activities like haircombing or dressing difficult (Donner and Kline 1993; Williams et al. 1996), thereby reducing quality of life. In 2007, SAN injury culminated in U.S. court settlements averaging $515,968, increasing to $15 million in recent years (Cesmebasi and Spinner 2015; Morris et al. 2008).

Reviewing the course of the SAN and its common variations will prove beneficial in updating our understanding of the nerve and determining which configuration should be taught in Medical Schools. Appreciating its variations and identifying vulnerable points along its course by means of preoperative imaging will also help prevent SAN injury.

This study provides an in-depth description of the SAN from its origin to its termination and discusses its surface anatomy, composition and questionable cranial nerve classification.

## Materials and Methods

An independent review of the literature on the spinal accessory nerve was carried out using Medline, PubMed and Google Scholar including results from January 1946 to May 2023. Terms searched for included combinations of ‘spinal accessory nerve’, ‘cranial nerve XI’, ‘anatomy’, ‘surface anatomy’, ‘variation’, ‘classification’, ‘anastomosis’ ‘nerve injury’, ‘nerve damage’ and ‘imaging’. All primary studies found to be discussing the spinal accessory nerve or its variations, including cadaveric dissections, surgical case studies and ultrasound findings, were considered. References within these articles were also used to discover further relevant papers, including articles prior to 1946. This yielded a total of 121 results. Exclusion criteria were papers referencing distortion of anatomy due to tumours, previous surgery or nerve injury. Nineteen articles were excluded on this basis. This left 102 articles which were stratified into those discussing gross anatomy, surface anatomy, fibre type and cranial nerve classification and miscellaneous information.

A dissection was carried out on two embalmed female cadavers (aged 68 and 90) to accompany this review. On one cadaver, a superficial and deep lateral neck dissection was carried out on one side within the combined parameters of the anterior and posterior triangles of the neck to demonstrate the relationship between the accessory nerve and the surrounding nerves, vasculature and musculature. A deep dissection on the posterior neck was carried out on the other cadaver. The laminae of C1-6 were removed and the dura and arachnoid were reflected with pins to expose the dorsal spinal rootlets and origin of the accessory nerve. A segment of occipital bone superior to the foramen magnum was drilled away and one side of the cerebellum was removed to expose the course of the intracranial accessory nerve.

The authors hereby confirm that every effort was made to comply with all local and international ethical guidelines and laws concerning the use of human cadaveric donors in anatomical research.

## The Spinal Accessory Nerve

### Origin and Intradural Course

Early human embryo studies have demonstrated the SAN arising from the spinal accessory nucleus, which extends craniocaudally along the C1-6 ventral horns (Pearson et al. 1938). However, the caudal termination of the nucleus is now disputed. Some authors report termination at C5 (Binder 2010; Kiernan 2009; Routal and Pal 2000) and others at C7 (Pearson 1938; Pearson et al. 1964)—a likely reflection of potential variability in this region.

**Fig. 1:**
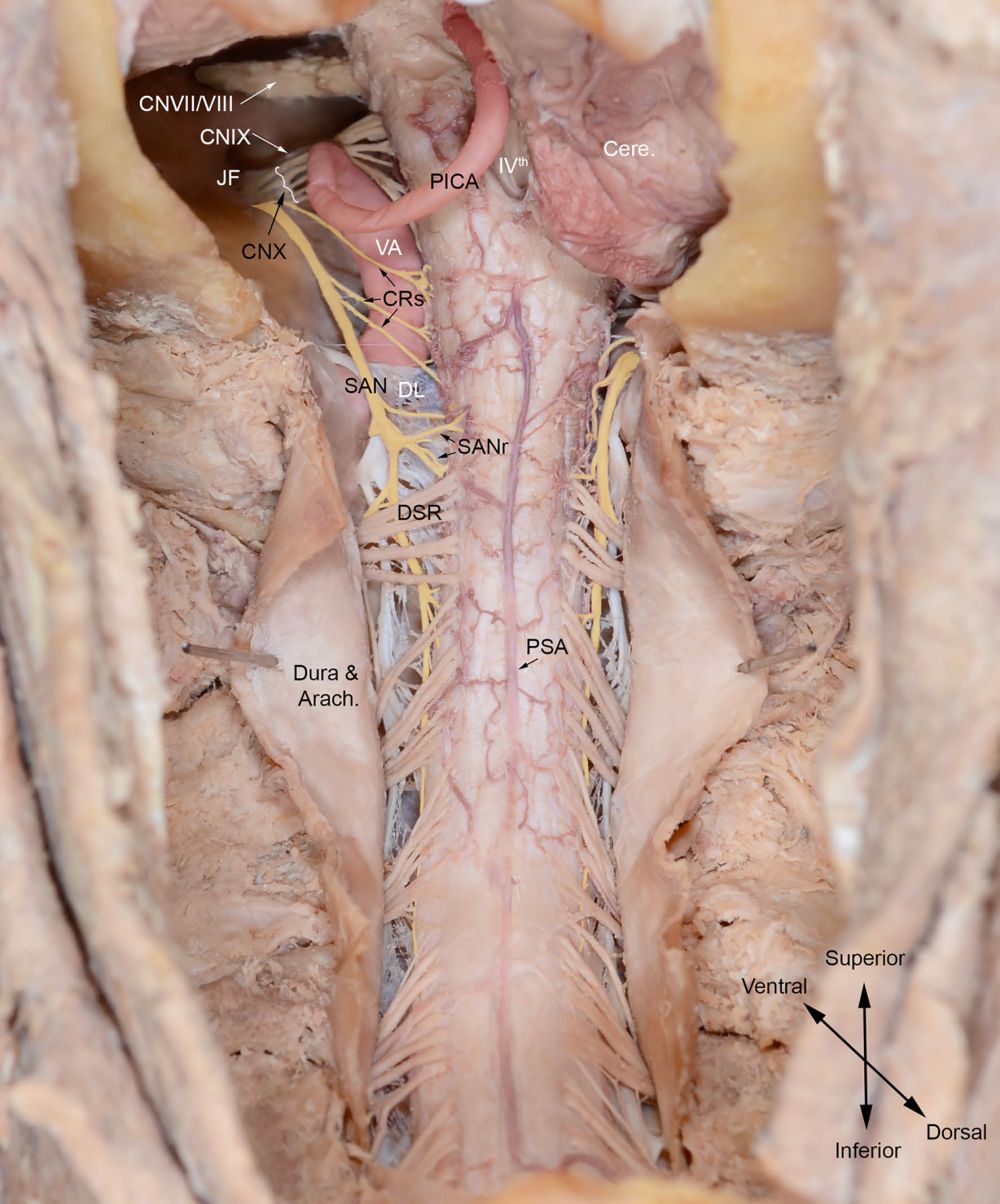
Dorsal spinal cord showing path of spinal accessory nerve (SAN) from its rootlets to the jugular foramen. Posterior inferior cerebellar artery (PICA) emerges between SAN and vagus nerve. *Cere* cerebellum, *CNVII/VIII* facial-vestibulocochlear nerve complex, *CNIX* glossopharyngeal nerve, *CNX *vagus nerve, *CRs* cranial roots of accessory nerve, *DL* denticulate ligament, *DSR* dorsal spinal rootlets, *Dura and Arach* dura and arachnoid mater, *JF* jugular foramen, *PICA* posterior inferior cerebellar artery, *PSA* posterior spinal artery. *SAN* spinal accessory nerve, *SANr* spinal accessory nerve rootlets, *VA* vertebral artery, *IV*th fourth ventricle

**Fig. 2:**
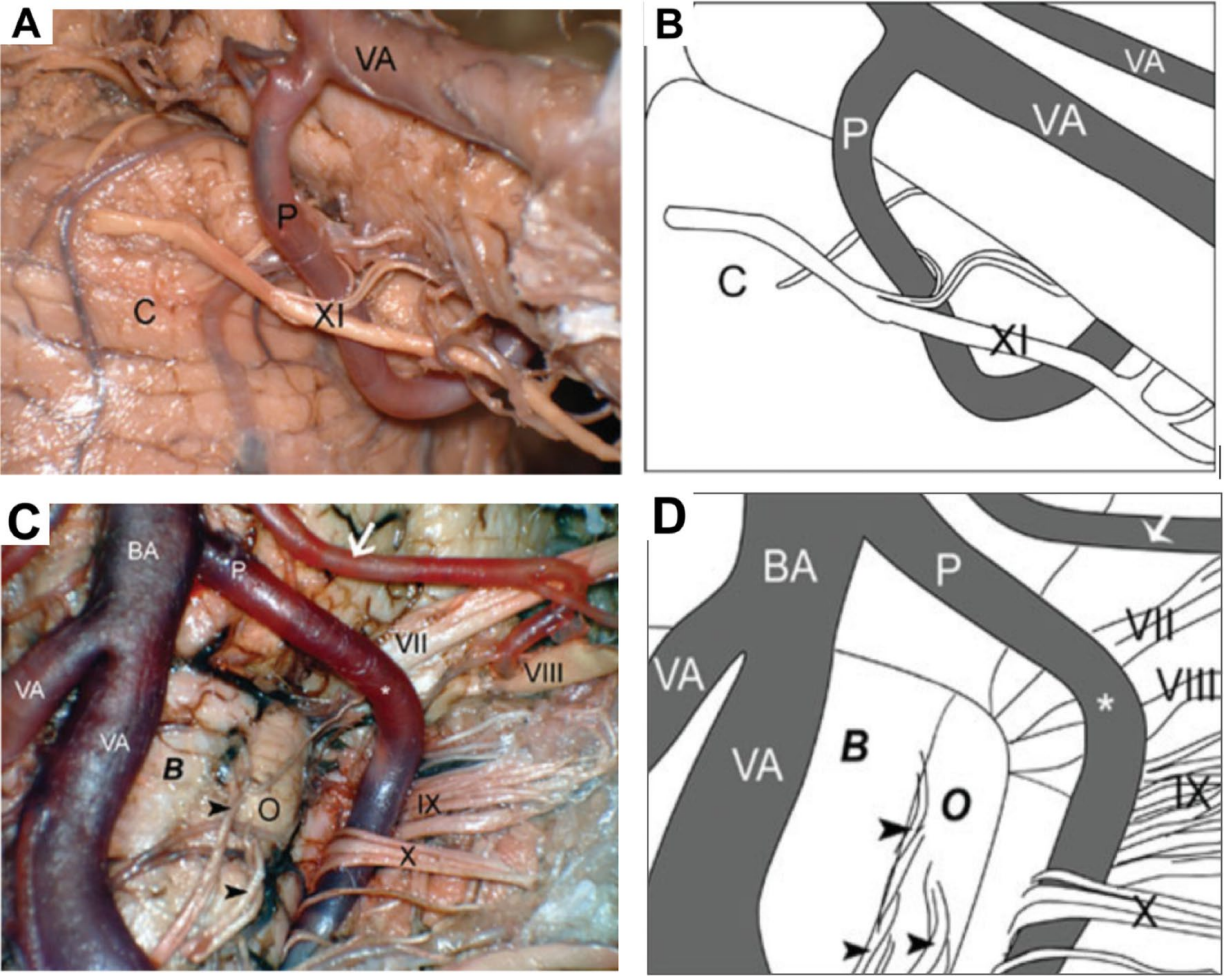
Photograph and Schematic of posterior inferior cerebellar artery penetrating spinal accessory nerve rootlets (**A** and **B**) and of posterior inferior cerebellar artery passing between glossopharyngeal and vagus nerves (**C** and **D**) (Saylam et al. 2007). *B* medulla oblongata (bulbous), *BA* basilar artery, *C* cerebellum, *O* olive, *P* posterior inferior cerebellar artery, *VA* vertebral artery, *VII* facial nerve, *VIII* vestibulocochlear nerve, *IX* glossopharyngeal nerve, *X* vagus nerve, *XI* spinal accessory nerve, *Arrow* anterior inferior cerebellar artery, *Arrowheads* rootlets of hypoglossal nerve

The nucleus gives rise to SAN rootlets (Fig. 1) which either course cranially before exiting the spinal cord or emerge directly from it (Saylam et al. 2009a, b). Classically, the rootlets are described as originating between C1-6 (Standring 2005). However, Saylam et al. found the inferior-most rootlet most commonly emerged from C3 (42.9%) and with progressively less common emergence from lower levels down as far as C5 (Saylam et al. 2009a, b). Established rootlets unite to form a common trunk that ascends cranially adjacent to the spinal cord, dorsal to the denticulate ligament and ventral to the dorsal spinal rootlets (Tubbs et al. 2008) (Fig. 1). There is no documented evidence of deviation from this relationship. The SAN then courses posterior to the vertebral artery (Saylam et al. 2007) and close to the Posterior Inferior Cerebellar Artery (PICA) (Fig. 1). PICA may be absent (Standring 2005) but when present it can course through the SAN rootlets (65%) (Fig. 2), between the vagus and SAN (20%) (Fig. 1) or between the glossopharyngeal and vagus nerves (12.5%) (Saylam et al. 2007) (Fig. 2). The SAN then courses intracranially via foramen magnum and exits through the jugular foramen (Fig. 1). Rarely, it can split and reform between these two foraminae (Tubbs, et al. 2017).

**Fig. 3:**
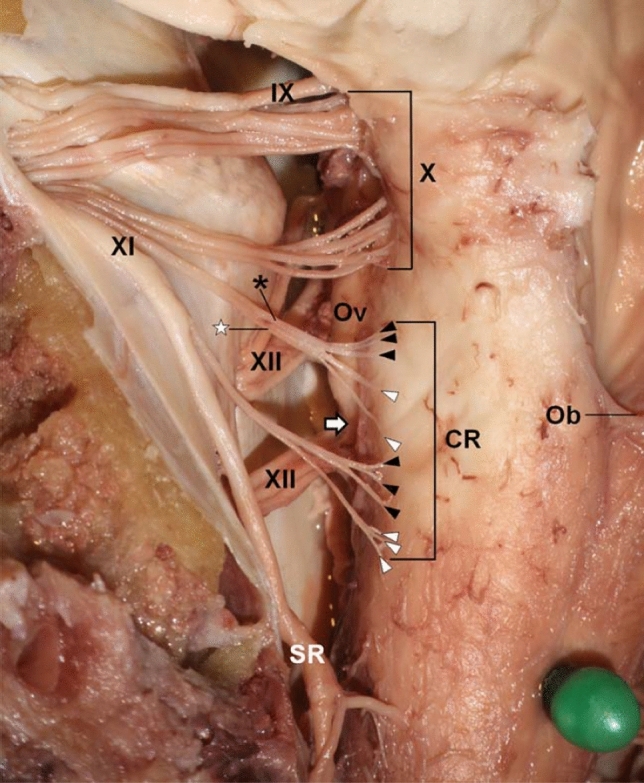
Accessory nerve cranial filaments (arrowheads), rootlets (asterisks) and roots (Liu et al. 2014a) (permission received from publisher for redistribution). *CR* cranial root, *Ob* obex, *Ov* olive, *SR* spinal root, *IX* glossopharyngeal nerve, *X* vagus nerve, *XI* accessory nerve, *XII* hypoglossal nerve, Uppermost and next rootlets are indicated by black and white colours respectively. Associated filaments are labelled with the same respective colours

The cranial root of the accessory nerve (present in 80% of cases (Tubbs et al. 2017)) emerges from the medulla as 2–9 rootlets (Liu et al. 2014a, b). These emerge singly (34%) or as a union of 2–4 filaments (66%) (Liu et al. 2014a, b) (Fig. 3). The cranial root ascends and fuses with the main trunk of the SAN before entering the jugular foramen; however, they remain as two distinct nerves microscopically (Ong et al. 2010). Conventionally, the cranial root fuses with the superior vagal ganglion at the jugular foramen (Lobstein’s anastomosis (Olry 1995)) and later provides branches supplying the pharynx, larynx and thoracic structures (Katsuta et al. 1997; Kim et al. 2003; Liu et al. 2015; Restrepo et al. 2015; Tubbs et al. 2014b).

**Fig. 4:**
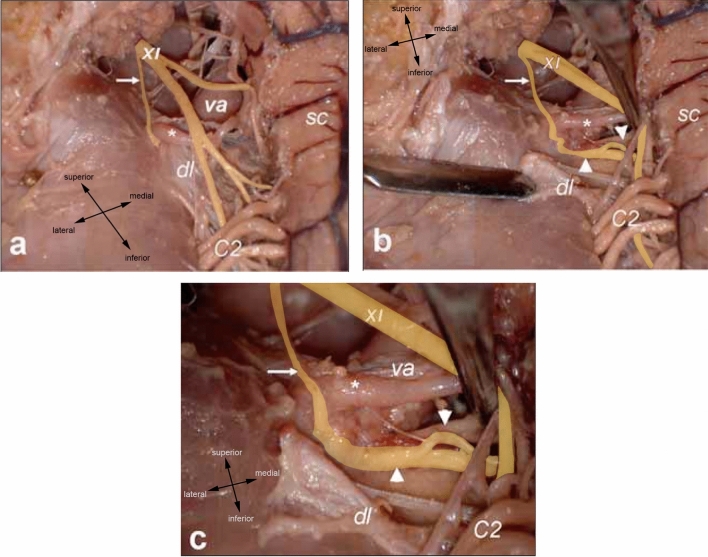
McKenzie branch linking the spinal accessory nerve to a ventral C1 rootlet. **a** shows accessory nerve in situ, **b** and **c** show accessory nerve reflected. Saylam et al. (2009a, b) (permission received from publisher for redistribution). *C2* dorsal C2 root, *dl* denticulate ligament, *SC* spinal cord, *va* vertebral artery, *XI* pinal accessory. *Asterisk* anterior spinal medullary artery, nerve, *Thin arrow* McKenzie branch, *Thick arrowheads* ventral C1 rootlets

During its ascent, the SAN undergoes numerous anastomoses with spinal rootlets of which the most clinically significant is the McKenzie branch where the SAN fuses with the ventral C1 rootlet (Fig. 4) (Saylam et al. 2009a, b). Its incidence was initially estimated at 50% (McKenzie 1954) but is now thought to vary between 3–7% (Friedman et al. 1993; Oh et al. 2003; Saylam et al. 2009a, b). The assumption that motor fibres coursed from the C1 ventral rootlet into the SAN along the McKenzie branch (McKenzie 1954) has been supported by electrical studies where stimulation of the C1 ventral rootlet resulted in SCM contraction (Friedman et al. 1993). Sometimes, the McKenzie branch causes a debilitating condition called Spasmodic Torticollis or Cervical Dystonia (Freckmann & Hagenah 1986). This involves intermittent, involuntary SCM contraction resulting in a sometimes painful tilting or pulling of the head (DystoniaSociety 2019). Treatment involves severing the McKenzie branch in an operation called Intradural Selective Rhizotomy (Oh et al. 2003).

**Fig. 5:**
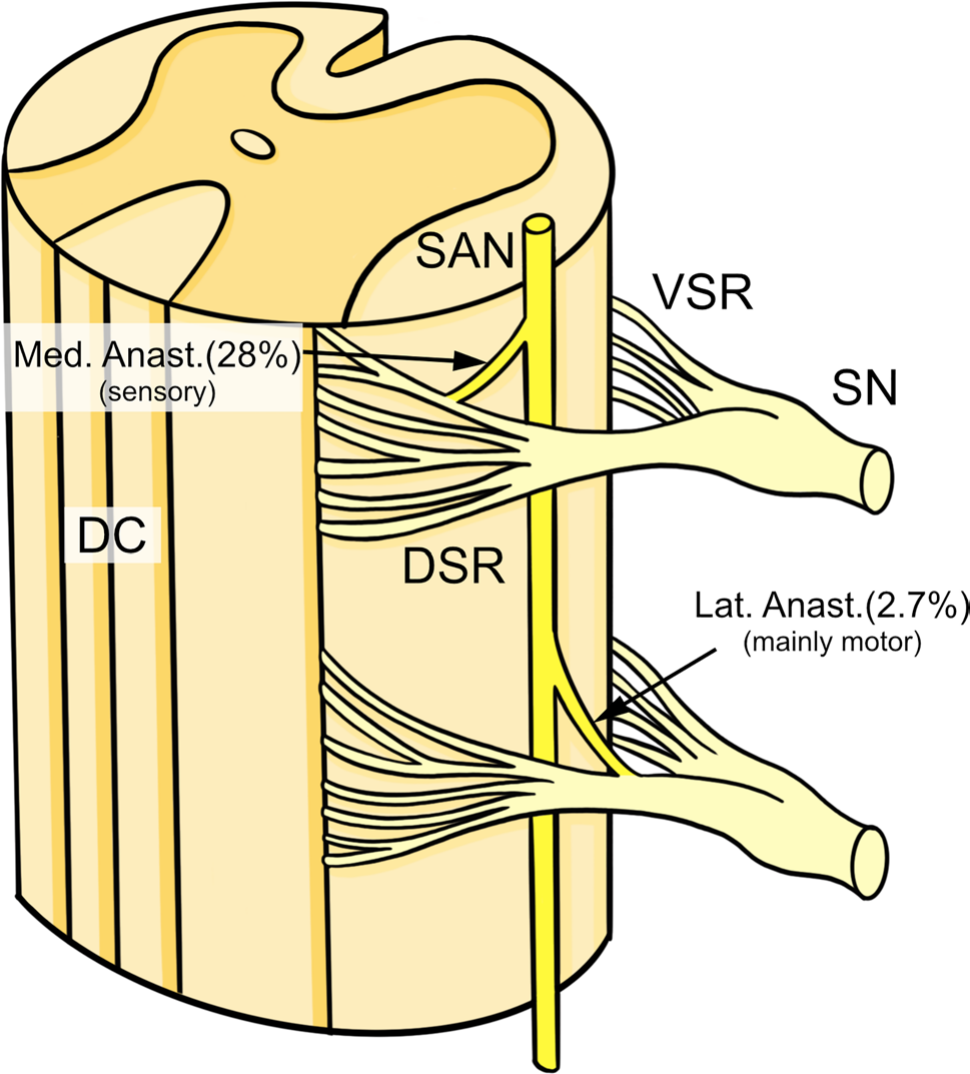
Dorsal root anastomoses occurring medial and lateral to the spinal accessory nerve (SAN) trunk. *DC* Dorsal Collumn, *DSR* dorsal spinal root, *Lat Anast* lateral anastomosis, *Med Anast.*  medial anastomosis, *SAN* spinal accessory nerve, *SN* spinal nerve, *VSR* ventral spinal root

Anastomoses also occur with dorsal rootlets. One study documents dorsal rootlet anastomoses occurring 28% medial to the SAN trunk and 2.7% laterally, where medial anastomoses were sensory and lateral anastomoses were predominantly motor (Tubbs et al. 2017) (Fig. 5). Anastomoses were most common between the dorsal C1 rootlet and the SAN (68%) (Ouaknine and Nathan 1973) and occurred at a decreased frequency every level caudal to this until C6 (Oh et al. 2001). The clinical significance of dorsal root anastomoses is currently unknown.

### Extradural Course

**Fig. 6:**
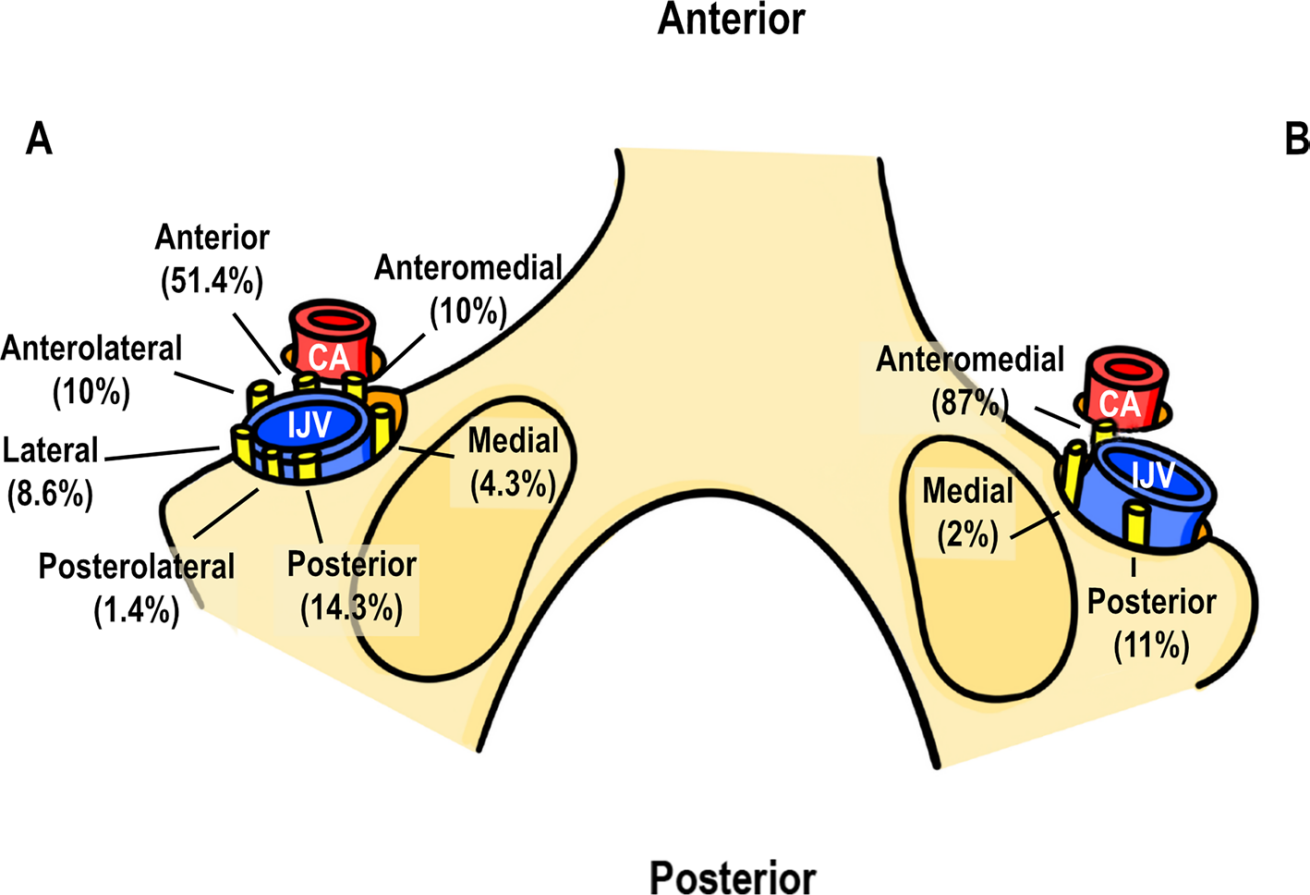
Relationship between spinal accessory nerve (SAN) and internal jugular vein (IJV) within the jugular foramen according to (**A**) Yigit et al. (2018) and (**B**) Saman et al. (2011) (exterior skull base views), *CA* carotid artery, *IJV* internal jugular vein

The course and variation of the SAN in the neck is well documented but its more complicated relationships within the jugular foramen continue to be studied. Within the jugular foramen, Saman et al. (2011) found the nerve coursing anteromedial to the Internal Jugular Vein (IJV) in the majority of cadavers (87%) and posterior to the IJV in a minority (11%). (Fig. 6B). Yigit et al. (2018) found the SAN most commonly coursing anterior to IJV (51.4%) and identified a greater number of variants than the former study with incidences ranging from 1–51% (Fig. 6A). Importantly, both studies concur that surgeons should be particularly vigilant when exploring the anterior jugular foramen to minimize risk of SAN injury.

**Fig. 7:**
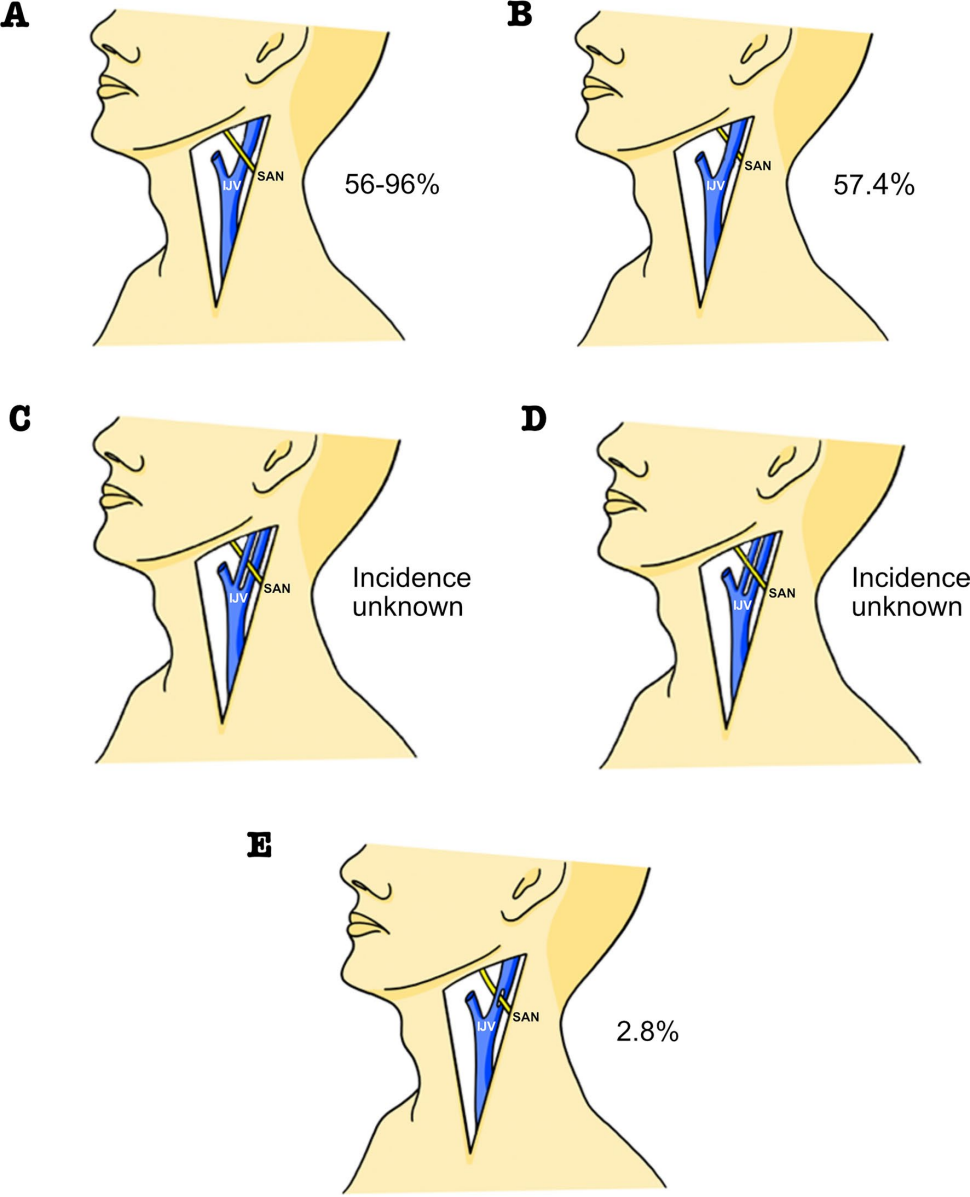
Relationship between the spinal accessory nerve and the internal jugular vein. **A** and **B** SAN crossing the IJV anteriorly and posteriorly; **C** and **D** SAN passing through/around IJV duplication; **E** SAN passing through IJV fenestration. *IJV* internal jugular vein, *SAN* spinal accessory nerve

The SAN emerges from the jugular foramen into the anterior triangle of the neck posterior to the styloid process (Bodner et al. 2002; Kim et al. 2003) and between the IJV and Internal Carotid Artery (ICA) (Leung et al. 2004). It most commonly crosses the IJV anteriorly (56–96%) (Caliot et al. 1989; Kierner et al. 2000; Soo et al. 1986), although one study reports posterior crossing as most frequent (57.4%) (Lee et al. 2009). Less commonly (2.8%), the SAN may pass through a fenestration in the IJV (Iseri et al. 2007; Lee et al. 2009). Rarely, the IJV may be duplicated (0.4% (Prades et al. 2002)) with the SAN traversing inbetween or around the two components (Ibrahim et al. 2016) (Fig. 7).

**Fig. 8:**
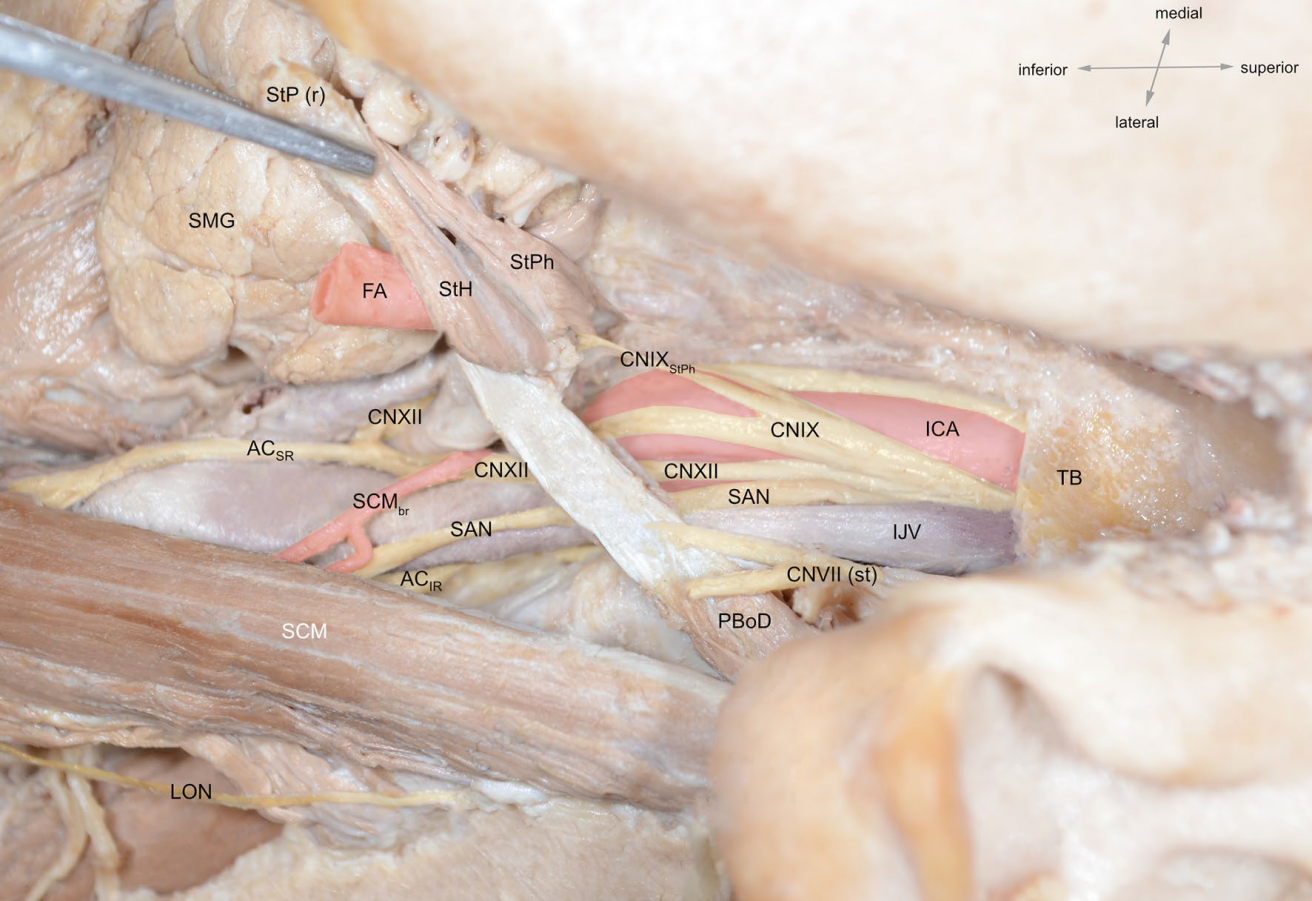
Cadaveric dissection showing anterior crossing of spinal accessory nerve (SAN) over internal jugular vein (IJV) and lateral positioning relative to IJV at the posterior belly of digastric muscle. *AC*_*IR*_ inferior root of ansa cervicalis, *AC*_*SR*_ superior root of ansa cervicalis, *CNVII(st)*  facial nerve stump, *CNIX* glossopharyngeal nerve, *CNIXStPh* glossopharyngeal nerve branch to stylopharyngeus muscle, *CNX* vagus nerve, *FA* facial artery, *ICA* internal carotid artery, *IJV* internal jugular vein, *LON* lesser occipital nerve, *PBoD* posterior belly of digastric muscle, *SAN* spinal accessory nerve, *SCM* sternocleidomastoid muscle, *SCMbr* sternocleidomastoid branch of occipital artery, *SMG* submandibular gland, *StH* stylohyoid muscle, *StPh* stylopharyngeus muscle, *StP(r)*  styloid process (reflected), *TB* temporal bone

Further caudally, the SAN courses deep to the posterior belly of digastric muscle. Here, it most commonly lies lateral to the IJV (70–96%) according to the majority of authors (Agur 2006; Ebling 1997; Hinsley and Hartig 2010; Hone et al. 2001; Kierner et al. 2000; Levy et al. 2001; Netter 2006; Cornelius et al. 1997; Taylor et al. 2013; Yigit et al. 2018) (Fig. 8). Some also report anterior (Agur 2006; Kierner et al. 2000; Netter 2006; Cornelius 1997; Saman et al. 2011; Yigit et al. 2018), posterior (Kierner et al. 2000; Saman et al. 2011; Standring 2005; Yigit et al. 2018) and medial (Ebling 1997; Kierner et al. 2000; Lee et al. 2009) locations as common.

The SAN continues inferiorly to innervate SCM. Commonly, branches originating from C1-4, including cervical plexus branches, course adjacent to and sometimes anastomose with the SAN at this point, supplying additional motor fibres to SCM (Kim et al. 2003; Leung et al. 2004; Tubbs et al. 2014b, 2011). The SAN courses deep to SCM and either penetrates the muscle or supplies a branch to innervate its deep surface (Guo et al. 2003; Kim et al. 2003; Lee et al. 2009). It is thought 8% of the SAN fibres terminate to supply SCM and 92% continue to supply the trapezius (Wang et al. 2014). Rarely, the SAN may split into superior and inferior branches prior to SCM, where the former supplies SCM and latter supplies trapezius (Brennan et al. 2015). Interestingly, one case documents an aberrant facial nerve branch innervating SCM, thought to arise due to developmental digastric and SCM fusion (Cvetko 2015).

### Termination

**Fig. 9:**
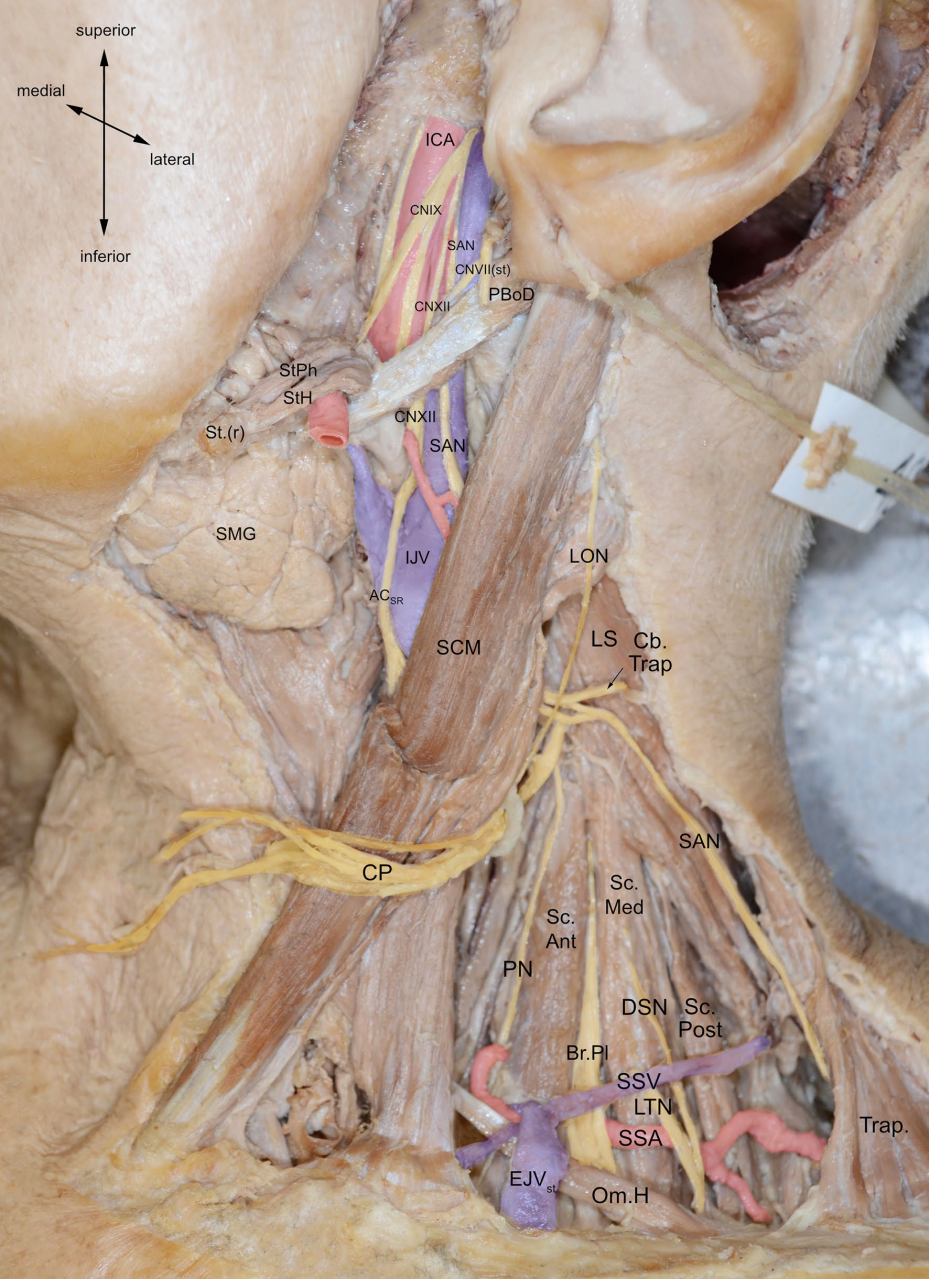
Extradural course and termination of the spinal accessory nerve (SAN). *AC*_*SR*_ superior root of the ansa cervicalis, *Br.Pl* brachial plexus, *CbTrap* cervical branch to trapezius muscle, *CNVII* facial nerve stump, *CNIX* glossopharyngeal nerve, *CNX* vagus nerve, *CP* cervical plexus, *DSN* dorsal scapular nerve, *EJV(st)*  external jugular vein stump, *ICA* internal carotid artery, *IJV* internal jugular vein, *LON* lesser occipital nerve, *LS* levator scapulae muscle, *LTN* long thoracic nerve, *OmH* omohyoid, *PBoD* posteror belly of digastric muscle, *PN* phrenic nerve, *SAN* spinal accessory nerve, *ScAnt* scalenus anterior muscle, *SCM* sternocleidomastoid muscle, *ScMed* scalenus medius muscle, *ScPost* scalenus posterior muscle, *SMG* submandibular gland, *SSA* suprascapular artery, *SSV* suprascapular vein, *St.(r)*  reflected styloid process, *StH* stylohyoid, *StPh* stylopharyngeus, *Trap* trapezius muscle

The SAN emerges from the posterior border of SCM between a third and half way down the muscle and around 7.5-9 cm superior to the clavicular head (Donner et al. 1993; Leung et al. 2004; Wang et al. 2014). It emerges superior to the Erb’s Point (Aramrattana et al. 2005; Durazzo et al. 2009; Salgarelli et al. 2009), where cervical plexus branches loop around the posterior border of SCM. The SAN then courses obliquely along the posterior triangle, deep to the investing layer of deep cervical fascia (Fig. 9) and superficial to the prevertebral fascia and levator scapulae (Kim et al. 2003; Leung et al. 2004) The average length of the SAN in the posterior triangle ranges from 4.4 to 8.2 cm (Durazzo et al. 2009; Mirjalili et al. 2012; Popovski et al. 2017; Wang et al. 2014), although its tortuous path means its length increases when stretched (Wang et al. 2014).

The SAN receives numerous anastomoses in the posterior triangle (Brown 2002), prompting Brown (2002) to coin the term “spinal accessory plexus”. Specifically, it can receive contributions from C2-3 branches (Williams et al. 1990), cervical plexus (predominantly the greater auricular nerve (Brown 2002)), brachial plexus, sympathetic chain and other cranial nerves (Brown 2002).

The SAN enters trapezius around 1.5 cm medial to the midclavicular line (Wang et al. 2014) and 4.5 cm from the clavicle (Aramrattana et al. 2005). It innervates all three divisions of the trapezius, whereas the upper and middle portions also receive contributions from C2-3 (Kim et al. 2003). This may explain findings of 90% muscle function being retained after SAN transection (Wang et al. 2014).

### Surface Anatomy and Intraoperative Identification

Appreciation of SAN anatomy relative to surrounding landmarks helps minimize risk of iatrogenic injury during procedures like lymph node biopsy and radical neck dissection.

**Fig. 10:**
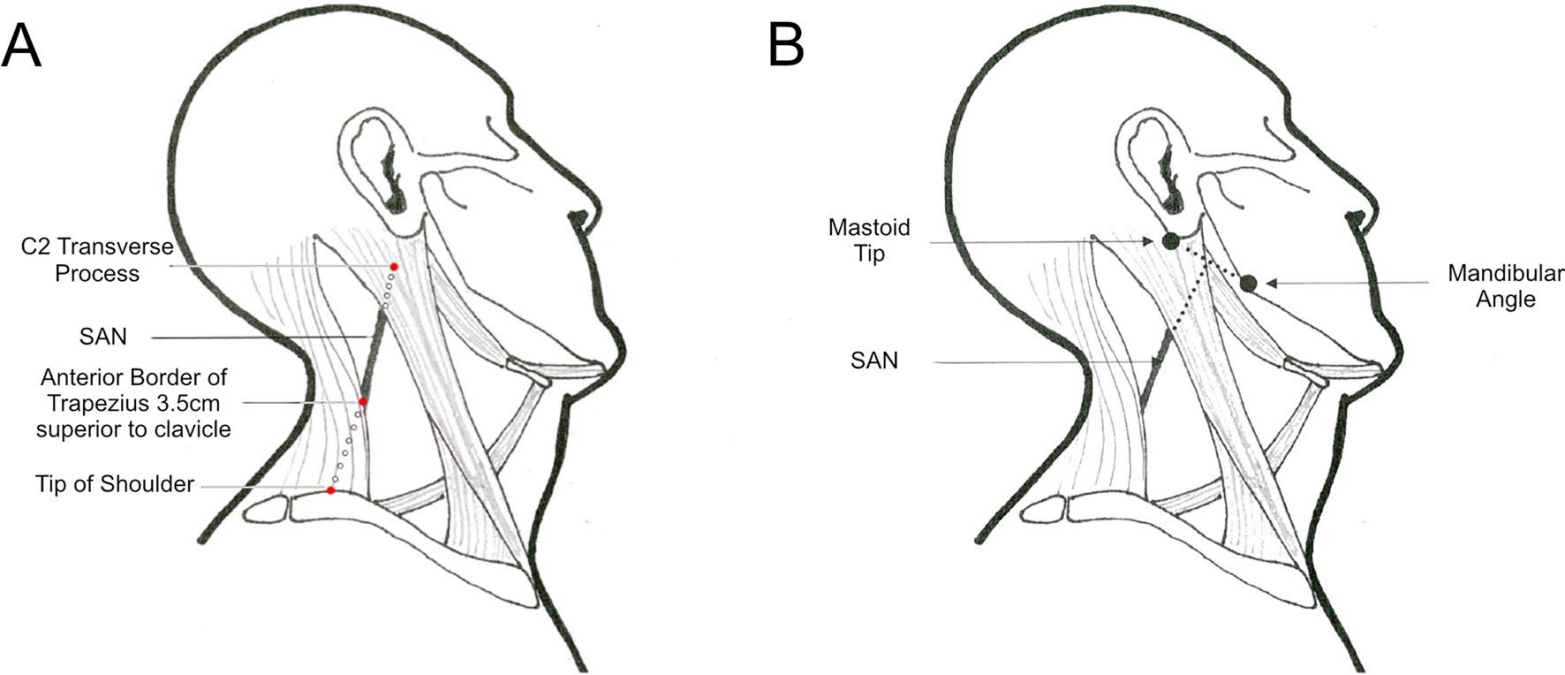
**A** Line joining C2 transverse process with tip of shoulder. **B** Perpendicular bisection of mastoid-mandibular angle line (modified from Chandawarkar et al. (2003), permission received from publisher for modification and redistribution)

The SAN emerges and exits the posterior triangle 2 cm above and 2 cm below an imaginary horizontal line transecting the thyroid notch (King and Motta 1983). The nerve’s path in the posterior triangle lies approximately along the course of a line joining the transverse process of C2 (just below the mastoid process) and either the tip of the shoulder (Donner et al. 1993; Kim et al. 2003; Sheen et al. 1997) or anterior border of trapezius 3-5 cm superior to the clavicle (Standring 2008). It can also be approximated by a perpendicular bisection of the mastoid-mandibular angle line (Fig. 10) (Chandawarkar et al. 2003).

The significance of where the bisection and posterior SCM intersect is disputed in literature. Some describe it as the emergence of SAN into the posterior triangle (Chandawarkar et al. 2003) whereas others describe it as Erb’s point (Baring et al. 2007; Salsche 1988). This highlights that these descriptions are merely guides and are not wholly accurate. Symes et al. comment that SAN variation within the posterior triangle precludes a working definition of surface anatomy (Symes and Ellis 2005). Clinical estimates of surface anatomy should therefore always be considered in conjunction with conventional neurophysiological methods and imaging.

**Fig. 11:**
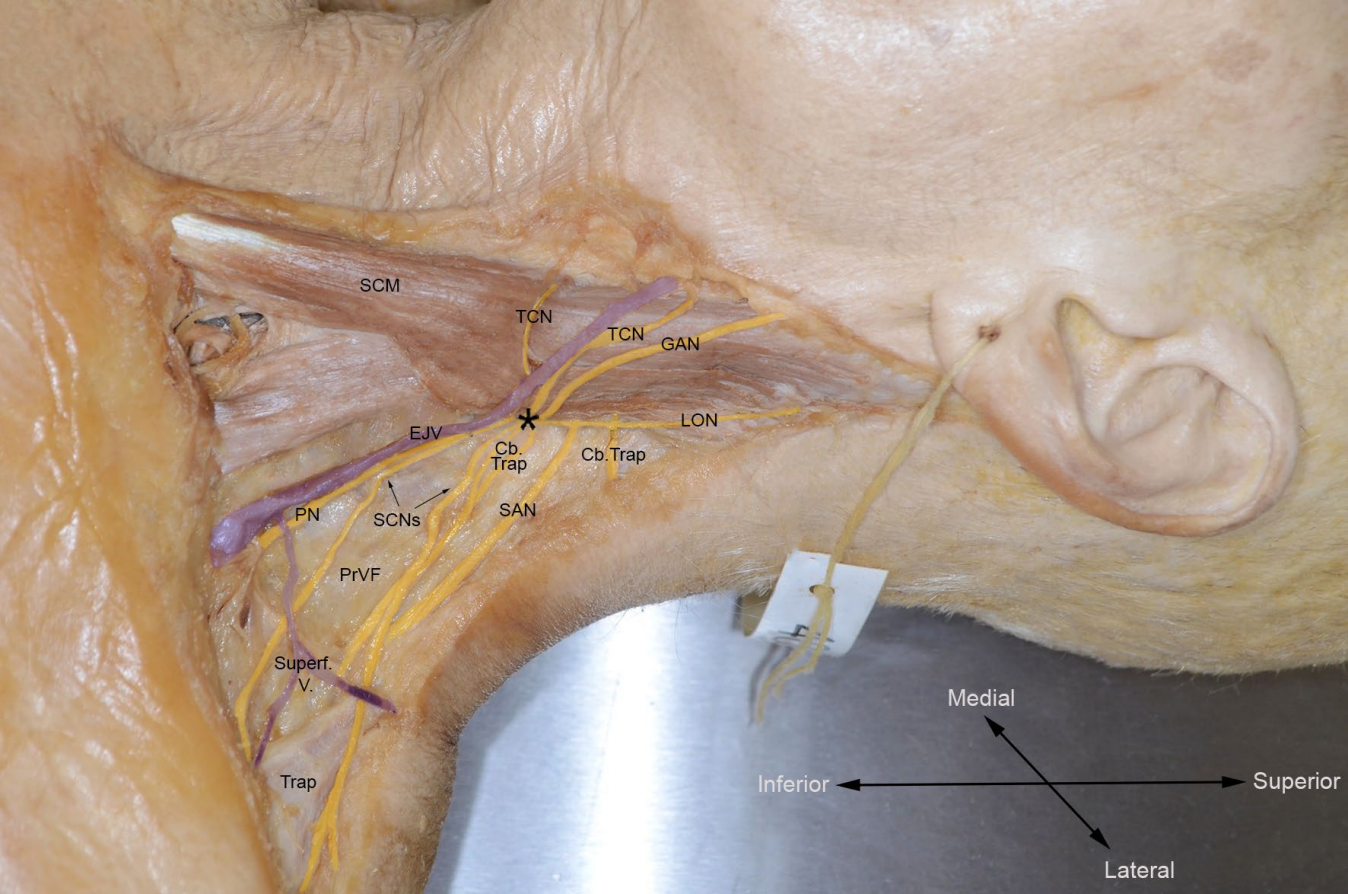
Spinal accessory nerve (SAN) exiting the posterior border of sternocleidomastoid superior to Erb’s point. *CbTrap* cervical branch to trapezius, *EJV* external jugular vein, *GAN* greater auricular nerve, *LON* lesser occipital nerve, *PN* phrenic nerve, *PrVF* prevertebral fascia, *SAN* spinal accessory nerve, *SCM* sternocleidomastoid muscle, *SCNs* supraclavicular nerves, *Superf. V.*  superficial veins, *TCN* transverse cervical nerve, *Trap* trapezius muscle, asterisk Erb’s point

Techniques for intraoperative SAN identification are invaluable during procedures like radical neck dissection. The most reliable and widely-used marker in surgery is the relationship between the SAN and Erb’s point (Chen et al. 2009; Durazzo et al. 2009; Guo et al. 2003; Hone et al. 2001; Leung et al. 2004; Popovski et al. 2017) (Fig. 11).

Most studies report SAN lying 0.9–2.0 cm superior to Erb’s point (Chaukar et al. 2006; Chen et al. 2009; Durazzo et al. 2009; Hill and Olson 1979; Hone et al. 2001; Mirjalili et al. 2012; Popovski et al. 2017; Soo et al. 1990, 1986) with a range of 0–3.9 cm (Aramrattana et al. 2005; Salgarelli et al. 2009). In the submandibular region, the SAN usually lies just anterior to the C2 transverse process (77.5%) (Durazzo et al. 2009). It emerges into the posterior triangle at a ratio of 0.34–0.53 craniocaudally along the posterior SCM border (Chaukar et al. 2006; Chen et al. 2009; Hale et al. 2010; Mirjalili et al. 2012) and enters trapezius at the junction between the middle and lower third of its anterior border (Hale et al. 2010). One author reports the latter relationship as one of the most reliable (Durazzo et al. 2009). In practice, neurophysiologic.

### Fibre Type

The SAN is conventionally taught as being composed solely of motor fibres innervating SCM and trapezius. This has long-since been recognized and demonstrated through electrical stimulation (Andrei and Marc 2017). However, it is now thought the SAN may be a mixed nerve with a sensory component (Pu et al. 2008; Tubbs et al. 2011), most likely due to anastomoses with the cervical plexus and dorsal spinal roots (Bremner-Smith et al. 1999; Caliot et al. 1989). Embryologically, the SAN is thought to develop as a mixed nerve and the degree to which the sensory component migrates to the dorsal roots determines the proportion of sensory fibres in the SAN. Bremner-Smith et al. (Bremner-Smith et al. 1999) found numerous unmyelinated fibres and small myelinated Ad fibres in the SAN, both of which are sensory and play a key role in nociception. Furthermore, neuronal cell bodies are observed along the SAN which are known to convey nociceptive stimuli from animal studies. Froriep’s ganglion, the existence of which is debated in humans (O'Rahilly and Müller 1984; O’Rahilly and Müller 2007; Pearson 1938; Streeter 1905; Tubbs et al. 2014a), is an embryological structure thought to ultimately form the nociceptive component of the SAN (Wetmore and Elde 1991). Its degeneration in 6–7 week embryos (Sensenig 1957) and visible absence in adults (Cho et al. 2015) is likely a result of programmed autolysis and the degree to which this occurs may explain the limited and variable presence of nociceptive pathways in adults (Cho et al. 2015).

### Is the Accessory Nerve Really a Cranial Nerve?

Galen, the early Greek physician, was first to document the presence of cranial nerves. He described seven pairs and defined them as ‘nerves which pass from the brain or brainstem as opposed to the spinal cord (Goss 1966).’ The accessory nerve was given its independent cranial nerve status in 1778 by Soemmering (Soemmerring 1778), whose classification system for cranial nerves is still used today (Eugene 1967; Shaw 1992; Simon et al. 2011). However, the cranial component wasn’t recognised until 1838 when Arnold published a series of elaborately drawn plates showing two components to the accessory nerve (Arnold 1838), that were later eluded to in the first edition of Gray’s Anatomy (Gray 1858).

The cranial root emerges from the medulla and ultimately fuses with the vagus nerve, whereas the SAN emerges from the upper spinal cord. Using Galen’s definition, the cranial root must fuse with the SAN to justify its cranial nerve classification or these structures should be considered as dorsal vagal rootlet and atypical intracranial spinal nerve respectively.

The existence of the eleventh cranial nerve is debated in literature with reports of the cranial root existing in all specimens (Liu et al. 2014a, b), the majority of cases (Tubbs et al. 2014a), variably (Wiles et al. 2007), the minority of cases (Ryan et al. 2007), or not at all (Lachman et al. 2002). Liu et al. (2014a, b) found the cranial root always fused with the SAN and was morphologically distinct from the vagus nerve. However, Lachman et al. (2002) found the cranial root always merged with the vagus nerve without SAN fusion. Such differences can perhaps be attributed to differing investigatory techniques and whether or not the cadaver was embalmed.

Studies describing cranial root existence in the minority of cases were based on small cadaver numbers (n = 11–15) (Lachman et al. 2002; Liu et al. 2014a, b; Wiles et al. 2007) whereas those proposing cranial root existence in the majority involved larger numbers (n = 25–43) (Liu et al. 2014a, b; Tubbs et al. 2014a). Since conventional cranial nerve definition draws upon morphology alone, demonstrating cranial root and SAN fusion in these larger studies could be argued as sufficient justification for the eleventh cranial nerve classification.

However, since the cranial root originates from the same nuclei as the vagus (nucleus ambiguus and dorsal motor nucleus) (Binder 2010; de Oliveira et al. 1985) and has similar functions to the vagus (Kim et al. 2003; Liu et al. 2015; Restrepo et al. 2015; Tubbs et al. 2014b), the cranial root could be considered as a dorsal vagal rootlet. Lachman et al. (2002) suggest that the need for meticulous dissection of the accessory nerve may be a reason for authors having accepted and re-referenced previous literature documenting the existence of the eleventh cranial nerve over the years. Interestingly, in Nkx2.9 gene knockout mice, cranial root development is inhibited whilst the SAN still forms (Dillon et al. 2005; Pabst et al. 1998, 2003; Schubert and Kaprielian 2001), further suggesting disjunction between the two components.

## Discussion

The course of the SAN is documented briefly in numerous articles, occasionally alluding to common variations in the posterior triangle, but few papers discuss variations along its entire course. This study offers a contemporary review of articles detailing an account of the nerve’s course from its origin to its termination, referring to literature on anatomical variations at each stage. This may be invaluable for surgeons operating close to the SAN, helping them appreciate its most common and possible configurations, thereby reducing risk of injury.

Due to its variability, classical surface anatomy merely approximates the nerve’s course. Pre-operative ultrasound tracing can result in quicker nerve localization but landmarks often become shifted following the raising of skin flaps (Salgarelli et al. 2009). Intraoperative landmarks can be reliably employed for SAN identification but these can be distorted by scarring from previous surgery (Kretschmer et al. 2001).

The discovery of nociceptive fibres in the spinal accessory nerve suggests the presence of an additional sensory component to the SAN. Their existence should validate the reclassification of the SAN from its conventional entirely motor nerve status to a mixed nerve. Direct injury to nociceptive fibres in SAN injury might explain post-injury shoulder pain—a symptom previously explained by traction neuritis secondary to shoulder droop (Bremner-Smith et al. 1999). This knowledge may change treatment plans for patients with post-operative pain and help provide more targeted management, which could be subject for further studies.

**Fig. 12:**
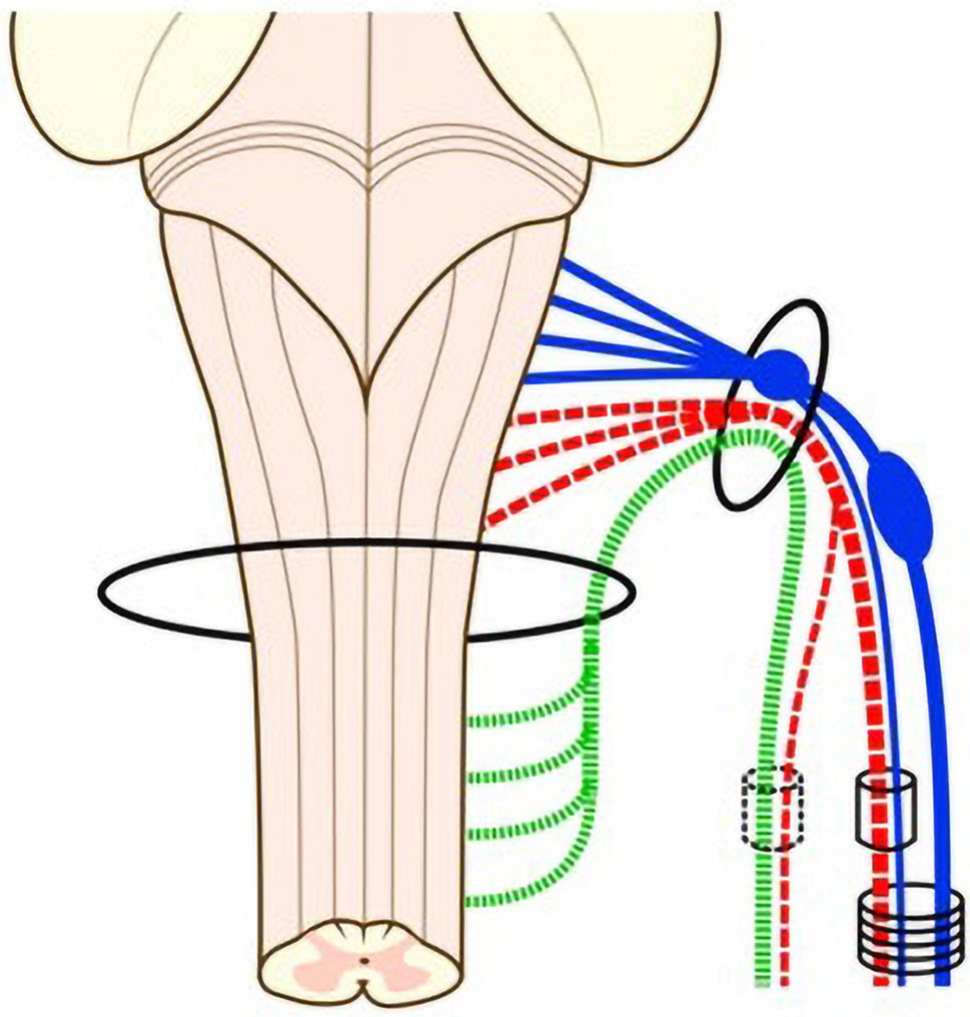
Commonest distribution of the spinal accessory nerve (SAN) cranial root Liu et al. (2014b) (permission received from publisher for redistribution). *Blue* vagus nerve. *Green* spinal root of accessory nerve, *Red* cranial root of accessory nerve

The exact cranial nerve classification of the accessory nerve is not definitive. The outcome of the debate also largely depends on how a cranial nerve is defined. The microscopic composition of the accessory nerve roots was analyzed in one Korean study, revealing that cranial root fibres most commonly split to combine with the vagus and with the SAN separately (Fig. 12) (Liu et al. 2014a, b). Despite the racial bias, these findings supported the current consensus that the accessory nerve should be classified as a cranial nerve with its two components considered functionally separate (Binder 2010; Standring 2005).

## Conclusion

The SAN has a variable course, especially at its origin and adjacent to the IJV. It is suggested that surgeons familiarize themselves with common variations of the SAN to best avoid nerve damage. The SAN should be redefined as a mixed nerve. Its cranial nerve classification is still largely debateable, however, microscopic studies suggest is should be considered as a cranial nerve with two components.

## Data Availability

Not applicable.
